# Chicken as a carrier of emerging virulent *Helicobacter* species: a potential zoonotic risk

**DOI:** 10.1186/s13099-025-00707-2

**Published:** 2025-05-21

**Authors:** Ahmed Samir, Hala M. Zaher

**Affiliations:** 1 Department of Microbiology, Faculty of Veterinary Medicine, Cairo University, Cairo, Egypt; 2 Department of Zoonoses, Faculty of Veterinary Medicine, Cairo University, Cairo, 12211 Egypt

**Keywords:** Chickens, *Helicobacter* spp., *cdtB* gene, Zoonosis, Public health

## Abstract

**Background:**

The research scope regarding *Helicobacter* species in chickens, other than *H. pullorum*, is largely overlooked. This study aimed to investigate the prevalence of emerging *Helicobacter* species in chickens and the occurrence of the virulence gene cytolethal distending toxin B (*cdtB*) among the identified *Helicobacter* species, referring to their public health significance.

**Methods:**

A total of 390 cloacal swabs were collected from 205 broilers and 185 layers. The swabs were pooled into 78 pools. DNA was extracted from these pools, followed by *Helicobacter* 16S rRNA gene PCR. Twenty pools positive for *Helicobacter* 16S rRNA were analyzed for *H. pylori* and *H. pullorum*, then *Helicobacter* 16S rRNA sequencing was performed on ten negative pools for *H. pullorum* and *H. pylori* to identify *Helicobacter* species. Subsequently, *cdtB* was investigated in the 20 pools positive for *Helicobacter*. Following that, partial DNA sequencing of one *H. pullorum* and one *H. brantae cdtB* gene from broiler and layer chickens, respectively, was carried out.

**Results:**

Overall, 25.6% of the examined pools were positive for *Helicobacter* spp., with 3 (7.3%) and 17 (45.9%) broiler and layer pools being positive, respectively. All three broiler pools were identified as *H. pullorum*; seven-layer pools were positive for *H. pullorum*, while *H. pylori* could not be detected. *Helicobacter* 16S rRNA sequencing of ten negative layer pools for *H. pullorum* and *H. pylori* revealed 6 *H. brantae*, 2 *H. kayseriensis*, 1 *H. winghamensis*, and 1 *Helicobacter* sp. Tul. The *cdtB* gene was found in 10 *H. pullorum*, 5 *H. brantae*, 1 *H. winghamensis*, and 1 *Helicobacter* sp. Tul. Phylogenetic analysis of *Helicobacter* 16S rRNA sequences and BLAST analysis of *H. pullorum* and *H. brantae cdtB* partial sequences underscore the public health importance of the obtained sequences.

**Conclusion:**

This study highlights that the occurrence of emerging virulent *Helicobacter* species in chicken feces poses a potential zoonotic relevance and public health risk.

## Background

*Helicobacter* is a Gram-negative, microaerophilic, spiral to curve-shaped bacterium isolated from the stomachs of mammals, including humans [1]. Based on phylogenetic analysis and ecological niches, this genus is broadly classified into two major subgroups: Gastric *Helicobacter* (GH) and Enterohepatic *Helicobacter* (EHH) species [2]. The most significant among gastric *Helicobacter* species is *Helicobacter pylori* (*H. pylori*), which has received priority attention worldwide due to its association with a variety of illnesses, such as peptic ulcer disease, gastric cancer, type B gastritis [3], and mucosa-associated lymphoid tissue (MALT) lymphoma [4]. The WHO has designated this bacterium as a Class I definite carcinogen [5] due to its significant role in most gastric malignancies. Later, *Helicobacter pullorum* (*H. pullorum*), an enterohepatic *Helicobacter* species [6], emerged and gained significant public health concern [7]. *H. pullorum* was identified as a new species by Stanley et al. [8] based on 16S rRNA phylogenetic analysis. This organism inhabits the intestinal tract of poultry and has been found in the liver and duodenum of asymptomatic birds, as well as in the liver and cecal contents of broiler chickens and laying hens suffering from vibrionic hepatitis [7, 9, 10]. In poultry slaughterhouses, *H. pullorum* has been found on chicken carcasses, possibly due to its high concentration in the cecum and subsequent contamination of raw chicken meat during slaughtering and evisceration [11]. Hence, it is considered an emerging foodborne zoonotic pathogen [7, 12]. Moreover, *H. pullorum* has been isolated from human patients suffering from gastroenteritis [13], chronic liver disorders [14], and even from clinically healthy persons [13].

Subsequently, other *Helicobacter* species have been recognized in poultry, although they remain beyond the primary research focus. For instance, *H. canadensis* was isolated from the feces of Barnacle geese (*Branta leucopsis*) and Canada geese (*Branta canadensis*) on the Atlantic coast of Europe [15], as well as from diarrheic and bacteremic patients [16, 17]. *H. pametensis* has been detected in the feces of wild birds [18]. Additionally, *Helicobacter anseris* (urease-positive) and *H. brantae* (urease-negative) have been identified in the feces of resident Canada geese in the United States [19]. Notably, several reports indicate that some enterohepatic *Helicobacter* species, including *H. hepaticus*, *H. bilis*, *H. cinaedi*, and *H. pullorum*, produce a well-characterized bacterial virulence element, the cytolethal distending toxin (CDT) [20–23]. CDT induces edema, cytoskeleton anomalies, and G2/M cycle arrest in host cells [24]. It is responsible for symptoms of infection, such as inflammation [25] and the development of diarrhea [22], and it has a potential role in intestinal carcinogenesis [26].

Although the conventional culture method is regarded as the gold standard test for *Helicobacter* detection, the delicate and fastidious nature of this pathogen makes it a challenging task [27]. This drives the development of molecular techniques like PCR, which do not rely on living bacteria as culture does and provide rapid and reliable results [28–31]. Since the majority of studies have focused on investigating *H. pullorum* in avian species [6, 9–11, 32–34], and knowledge is scarce regarding other *Helicobacter* species in poultry, the current study was conducted to investigate the prevalence of emerging *Helicobacter* species among broiler and layer chicken cloacal swabs, as well as to detect the *cdtB* virulence gene among the retrieved *Helicobacter* species to highlight their public health significance.

## Methods

### Sample collection

A total of 390 cloacal swabs were collected from 205 broilers and 185 layers at commercial poultry farms in Giza, Fayoum, and Qalyubia governorates, Egypt, from March 2023 to October 2023. Both apparently healthy (n = 210) and diseased chickens (n = 180) were included in this study. Each swab was placed in a tube containing 2 mL of normal saline (0.9%) and transported to the laboratory of Microbiology Department, Faculty of Veterinary Medicine, Cairo University, within two hours. Upon arrival, the cloacal swabs were processed in pools, each consisting of five cloacal swabs [35].

### DNA extraction

The pooled cloacal swabs were vigorously vortexed, and DNA was extracted from 78 pools using the QIAamp Fast DNA Stool Mini Kit (Qiagen, Germany) according to the manufacturer's instructions. All extracted DNAs were stored at -20 °C for further PCR analysis.

### Molecular identification of *Helicobacter* 16S rRNA gene

For genus confirmation through *Helicobacter* 16S rRNA amplification, the following oligonucleotide primers were used: (F:5-GGCTATGACGGGTATCCGGC-3 & R:5-GCCGTGCAGCACCTGTTTTC-3), as described by Moyaert et al. [36], 5 μL of DNA template was mixed with 12.5 μL of Cosmo PCR RED Master Mix (Willowfort, UK), 1 μL of each primer (10 pmol), and 5.5 μL of nuclease-free water. The PCR reaction was carried out under the following conditions: 95 °C for 5 min, followed by 45 cycles of 95 °C for 30 s, 65 °C for 30 s, 72 °C for 30 s, and a final extension at 72 °C for 10 min.

### Molecular detection of *H. pullorum* and *H. pylori*

Twenty pools positive for *Helicobacter* spp. were subjected to polymerase chain reaction using oligonucleotide primers targeting the *H. pullorum* 16S rRNA gene and the *H. pylori* phosphoglucosamine mutase gene (*glmM*) according to the protocol described by Elrais et al. [6]. The PCR conditions for the *H. pullorum* 16S rRNA gene were as follows: 94 °C for 5 min, followed by 35 cycles of denaturation at 94 °C for 1 min, annealing at 60 °C for 1 min, and extension at 72 °C for 1 min, with a final extension at 72 °C for 10 min. For the *H. pylori glmM* gene, the PCR mixture was preheated at 94 °C for 5 min, followed by 35 cycles of denaturation at 94 °C for 1 min, annealing at 55 °C for 1 min, and extension at 72 °C for 1 min, with a final extension at 72 °C for 10 min.

### Partial *Helicobacter* 16S rRNA gene sequencing and phylogenetic analysis

**Fig. 1:**
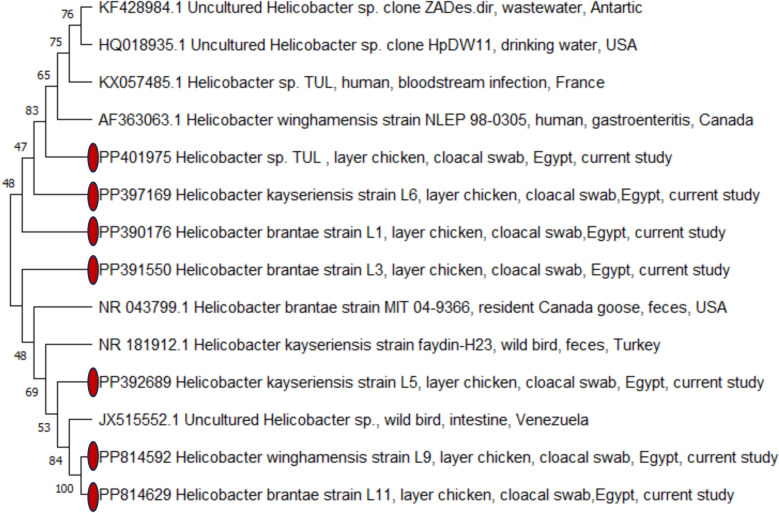
A phylogenetic bootstrap consensus tree was inferred via a neighbor-joining approach using MEGA 7 software to show the evolutionary history and genetic relatedness between *Helicobacter* 16S rRNA partial sequences obtained in this study and *Helicobacter* strains retrieved from the GenBank records

To identify *Helicobacter* species in ten pools of layer hens positive for *Helicobacter* 16S rRNA but negative for *H. pylori* and *H. pullorum*, partial 16S rRNA sequencing was performed. Amplicons were purified using a QIAquick purification kit (Qiagen, Germany) and sequenced with an ABI 3500 Genetic Analyzer (Applied Biosystems, USA). The recovered *Helicobacter* sequences in this study were aligned against those retrieved from birds, humans, and the environment available on the GenBank database to understand the genetic relatedness of our sequences. ClustalW multiple alignment was conducted using BioEdit software version 7.0.9, while MEGA 7 software was used to construct a phylogenetic tree via a neighbor-joining approach where a bootstrap consensus tree was obtained with 500 replicates (Fig. 1).

### Nucleotide sequence accession numbers

The partial *Helicobacter* 16S rRNA sequences generated in this study from layer chickens were *Helicobacter brantae* (accession no. PP390176, PP391029, PP391658, PP391550, PP814627, and PP814629), *Helicobacter kayseriensis* (accession no. PP392689 and PP397169), *Helicobacter winghamensis* (accession no. PP814592), and *Helicobacter* sp. TUL (accession no. PP401975).

### Molecular investigation of virulence gene cytolethal distending toxin B (*cdtB*) among *Helicobacter* species

**Fig. 2:**
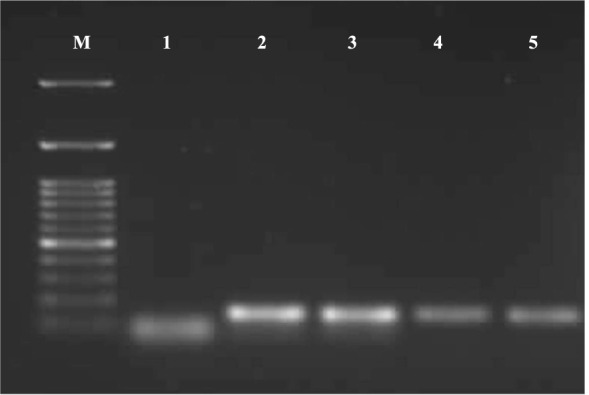
PCR amplification of the *cdtB* virulence gene in *Helicobacter* species. Lane M: DNA ladder (100 bp); lane 1: negative control; lanes 2, 3, 4, and 5: positive samples showed specific bands at 148 bp

Twenty positive pools for *Helicobacter* spp. were investigated for cytolethal distending toxin B (*cdtB*) as previously reported by Laharie et al. [37]. The primer sequence was F1: GTCTTTTGAGTGGATTGGATTCT and R2: CACTCCGGGTGCTTGTGTAT. Briefly, a 25 μL reaction mixture was created for each sample by adding 12.5 μL of COSMO PCR RED Master Mix (Willowfort, UK), 1 μL (10 pmols) of each primer, 5 μL of DNA template and 5.5 μL of PCR-grade water. The PCR thermal profile was as follows: 5 min initial denaturation at 94 °C, followed by 40 cycles of denaturation at 94 °C for 30 s, annealing at 60 °C for 60 s, and extension at 72 °C for 30 s, with a final extension at 72 °C for 10 min. The amplified amplicons were photographed after electrophoresis in 0.5 Tris–borate-EDTA using 1.5% agarose gel stained with ethidium bromide solution, where specific bands were detected at 148 bp (Fig. 2).

### Partial DNA sequencing of *Helicobacter cdtB* virulence gene

The PCR products of one *H. pullorum* and one *H. brantae cdtB* gene obtained from broiler and layer chickens, respectively, were purified via a QIAquick purification kit (Qiagen, Hilden, Germany). Afterwards, sequencing was carried out using Big Dye Terminator V3.1 kit (Thermo Fisher, USA) in an ABI 3500 Genetic Analyzer (Applied Biosystems, USA).

### Nucleotide sequence accession numbers

Partial sequences of the *H. brantae* and *H. pullorum cdtB* gene were submitted to GenBank and deposited in the GenBank database with the following accession numbers: PP486371 for *H. pullorum cdtB* from broilers and PP486372 for *H. brantae cdtB* from layers.

### Sequence identity BLAST analysis

The obtained *H. pullorum* and *H. brantae cdtB* partial sequences from broilers and layers, respectively, were compared with *H. pullorum cdtB* strains isolated from human cases available on GenBank using the NCBI website via BLAST analysis to display the identity percentage between our sequences and those of humans to clarify the public health impact of such sequences.

### Statistical analysis

The modified Wald method was used to calculate the 95% confidence interval (CI) of an overall prevalence value using the GraphPad QuickCalc online tool. The chi-square (χ^2^) test was utilized to evaluate the correlation between the examined chickens' clinical status and *Helicobacter*'s prevalence using IBM SPSS Statistics for Windows, software version 29.0.2.0 (Armonk, NY: IBM Corp). The result was considered statistically significant when the *p*-value was less than 0.05.

## Results

### Prevalence of *Helicobacter* spp. among the examined chickens

Overall, *Helicobacter* spp. was detected in 20 out of 78 examined pools (25.6%; 95% CI 17.19–36.38), where 7.3% and 45.9% of the investigated broilers and layers were positive, respectively (Table 1). Regarding the clinical status of the examined chickens, *Helicobacter* spp. was found in 18 (42.9%) apparently healthy and 2 (5.6%) diseased chickens, as shown in Table 2. The difference in the prevalence of *Helicobacter* among apparently healthy and diseased chickens was significant (*p*-value = 0.000169).

**Table 1:** Prevalence of *Helicobacter* spp. among broiler and layer chickens

Chicken species	No. of examined pools	Positive pools	
		No	%
Layers	37	17	45.9
Broilers	41	3	7.3
Total	78	20	25.6

**Table 2:** Prevalence of *Helicobacter* spp. among apparently healthy and diseased chickens

Chicken clinical status	No. of examined pools	Positive pools	
		No	%
Apparently healthy	42	18	42.9
Diseased	36	2	5.6
Total	78	20	25.6

### Prevalence of *H. pylori* and *H. pullorum* among the examined chickens

*H. pullorum* was found in 10 (12.8%) out of 78 examined pools where all three pools of broilers (3/41; 7.3%) were identified as *H. pullorum* and seven pools of layers (7/37; 18.9%) were positive for *H. pullorum*, whereas *H. pylori* could not be recognized among the examined broilers and layers in this study.

### Prevalence of *Helicobacter* species other than *H. pullorum* in layer chickens

The partial 16S rRNA sequencing of 10 pools of layers positive for *Helicobacter* 16S rRNA but negative for *H. pylori* and *H. pullorum* revealed *Helicobacter brantae* (n = 6; 16.2%), *Helicobacter kayseriensis* (n = 2; 5.4%), *Helicobacter winghamensis* (n = 1; 2.7%), and *Helicobacter* sp. TUL (n = 1; 2.7%), as shown in Table 3.

**Table 3:** Prevalence of *Helicobacter* species other than *H. pullorum* in layer chickens

Chicken species	No. of examined pools		*Helicobacter* spp.			Total
		*H. brantae*	*H. kayseriensis*	*H. winghamensis*	*Helicobacter* sp. TUL	
Layers	37	6 (16.2%)	2 (5.4%)	1 (2.7%)	1 (2.7%)	10 (27%)

### Phylogenetic analysis of partial *Helicobacter* 16S rRNA sequences

The phylogenetic analysis exhibited that *Helicobacter* spp. 16S rRNA sequences retrieved from layer hens in this study were closely related to *Helicobacter* strains isolated from wild birds as well as there was a genetic relationship between our sequences and those obtained from humans and the environment (Fig. 1).

### The distribution of *cdtB* virulence gene among *Helicobacter* species

Regarding *H. pullorum*, seven from apparently healthy layers and three from broilers (one and two from apparently healthy and diseased chickens, respectively) were positive for *cdtB* gene. For *H. brantae*, *cdtB* could be found in five *H. brantae* from apparently healthy layers. In addition, one *H. winghamensis*and one *Helicobacter* sp. Tul from apparently healthy layers carried *cdtB*, whereas two *H. kayseriensis* from apparently healthy layer hens were negative for *cdtB*, as exhibited in Table 4.

**Table 4:** The distribution of the cytolethal distending toxin B (*cdtB*) virulence gene among the obtained *Helicobacter* species

*Helicobacter* species	Number	Chicken species	Clinical status	*cdtB*
*H. pullorum*	7	Layers	Apparently healthy	+ ve
*H. pullorum*	1	Broilers	Apparently healthy	+ ve
*H. pullorum*	2	Broilers	Diseased	+ ve
*H. brantae*	5	Layers	Apparently healthy	+ ve
*H. brantae*	1	Layers	Apparently healthy	− ve
*H.kayseriensis*	2	Layers	Apparently healthy	− ve
*H. winghamensis*	1	Layers	Apparently healthy	+ ve
*Helicobacter* sp. Tul	1	Layers	Apparently healthy	+ ve

### Sequence identity BLAST analysis

The similarity ratios between the obtained *cdtB* sequences of *H. pullorum* and *H. brantae* of broilers and layers, respectively, in the current study and those of *H. pullorum cdtB* of public health significance as determined by BLAST analysis are displayed in Table 5.

**Table 5:** The identity percentage between the obtained *Helicobacter cdtB* partial sequences in this study and *H. pullorum cdtB* strains deposited in the GenBank of public health significance

Sequence	GenBank ID	Isolation source	% identity
PP486371 *H. pullorum* *cdtB* sequence, broiler chickens	JX434699.1	Patients with gastroenteritis	99.07
*H. pullorum cdtB* sequence, broiler chickens	JX434696.1	Patients with gastroenteritis	99.07
	AY394475.1	Liver of patient with cirrhotic Hepatitis C and without hepatocellular carcinoma	99.07
PP486372 *H. brantae cdtB* sequence, layer chickens	JX434699.1	Patients with gastroenteritis	98.68
	JX434698.1	Patients with gastroenteritis	98.68
	JX434697.1	Immunodeficient patient with diarrhea	99.48

## Discussion

Currently, understanding the epidemiological aspects of zoonotic *Helicobacter* species is a subject of great interest among researchers and scholars worldwide [7, 38–40]. In the present study, the prevalence of *Helicobacter* species 16S rRNA in cloacal swabs from the examined chickens (25.6%) was higher than that reported by Elrais et al. [6] (12%) in chicken meat in Egypt and García-Amado et al. [41] (5%) in the feces of wild birds in Venezuela, but lower than that detected by Fox et al. [19] (40.2%) in the feces of resident Canada geese in the Greater Boston region. Notably, *Helicobacter* spp. was found in apparently healthy chickens at a higher prevalence than in diseased ones. There was a significant difference between the two groups, suggesting that apparently healthy chickens may serve as a potential reservoir for *Helicobacter* species, raising public health concerns.

Regarding *H. pylori*, all broilers and layers in this study were negative for *H. pylori*. However, Elrais et al. [6] detected *H. pylori* in 300 broiler chicken samples (meat and giblets) with a prevalence rate of 5.33% (16/300) and El Dairouty et al. [42] revealed that 5% (1/20) of raw poultry meat samples were positive for *H. pylori*. Almashhadany et al. [43] found that 18 (13.8%) of 260 raw chicken meat samples tested positive for *H. pylori*, with 11 (15.7%) and 7 (11.7%) from the thigh and breast, respectively, while Asadi et al. [44] identified *H. pylori* in raw chicken meat samples at a rate of 15%. In the study conducted by Hamada et al. [45], 7 (7.78%) of 90 chicken samples were positive for *H. pylori*, including 6.67% of chicken meat and gizzards and 10% of liver. The detection of *H. pylori* in chicken meat in previous studies might be attributed to contamination by the hands of butchers, veterinarians, and abattoir workers during handling, preparation, and packaging, as well as the use of unclean water for washing chicken carcasses [6]. This could explain why *H. pylori* was not identified in cloacal swabs from the examined chickens in the present study.

For *Helicobacter pullorum*, the overall prevalence in the examined chickens was 12.8%. Our findings were higher than those of Hassan et al. [46], who detected *H. pullorum* in 7% (21 out of 300) of chicken cloacal swabs. Many studies have focused on investigating *H. pullorum* in chicken meat, breast, thigh, liver, ceca, and wings [6, 9–11, 46–48] rather than cloacal swabs. For instance, *H. pullorum* was detected in 32.29% and 10.15% of broiler chicken caeca and colon, respectively, in Turkey [49]; in caeca (7.5%), liver (5%) and thigh (2.5%) of broiler chickens with gastroenteritis in Aradabil [50]; and in 41% of broiler chicken caeca in Iran [10]. *H. pullorum* was also identified in the cecum, colon, jejunum, and liver of broiler chickens in Belgium with a prevalence of 33.6%, 31.8%, 10.9%, and 4.6%, respectively [9]; 24.72% of broiler and village chickens in Malaysia [51]; 23.52% of chicken meat in Lisbon [11]; and 30% of tested chicken wings in Iran [52]. Furthermore, the commercial chicken eggs are also believed to be infected with this pathogen [7]. In Egypt, *H. pullorum* was isolated from the examined baladi hen’s eggshells and egg contents in a percentage of 3.33% for each [53], as well as 10% and 5% of Baladi and poultry farm hen’s eggshells were contaminated with *H. pullorum*, respectively [54]. The occurrence of *H. pullorum* in cloacal swabs suggests that this pathogen may be transmitted to chicken carcasses via cross-contamination during the slaughtering process [7], and hens' feces may spread *H. pullorum* to eggs [54]. As *H. pullorum* is directly transmitted to humans through fecal contamination [8], poultry excreta represent a potential source of infection to various human populations, particularly slaughterhouse workers, farmers, and housewives [7]. It was noted that the prevalence of *H. pullorum* was higher in layers (18.9%) than in broilers (7.3%) in this study, whereas a study conducted in Iran found a higher occurrence in broilers (30%) compared to laying hens (13.3%) [55]. From a public health perspective, *H. pullorum* is an emerging zoonotic pathogen responsible for life-threatening human infections [12]. It has been detected in stool samples from human patients suffering from gastroenteritis, with a prevalence of 6% in Aradabil [50], as well as in the feces from patients with gastrointestinal disease (4.3%) and clinically healthy individuals (4.0%) in Belgium [13]. Furthermore, *H. pullorum* is associated with recurrent diarrheal illness [56] and it is implicated in cholelithiasis, cirrhosis [14], and gallbladder cancers [57, 58]. This association is attributed to the pathogen's ability to tolerate high bile stress [12]. Additionally, it has been recognized in patients with Crohn's disease [37, 59].

In the current work, partial *Helicobacter* 16S rRNA gene sequencing revealed other *Helicobacter* species in layer chickens. *H. brantae* was the most prevalent species identified in cloacal swabs of layers (16.2%), followed by *H. kayseriensis* (5.4%), *H. winghamensis* (2.7%), and *Helicobacter* sp. TUL (2.7%). To the best of our knowledge, *H. brantae*, *H. kayseriensis*, *H. winghamensis*, and *Helicobacter* sp. TUL were detected for the first time in layer chickens in this study. Regarding *H. brantae*, Kaakoush et al. [60] found this species in 64.5% of broiler chicken fecal samples. This urease-negative *Helicobacter* species was first identified in the feces of seven resident Canada geese within the Greater Boston area [19], and it was detected at a low incidence in tropical terrestrial wild birds in Venezuela [41]. Although the pathogenesis of this bacterium remains unclear, the occurrence of *H. brantae* in chickens may pose a zoonotic risk, potentially infecting other species of birds and mammals [19]. *H. kayseriensis* was recognized by Aydin et al. [61] in the feces of urban wild birds in Turkey. Moreover, *H. kayseriensis* was the most common species (28.57%) isolated from Taiwan's Yanshui and Donggang rivers [2]. *H. winghamensis* was first discovered in patients with gastroenteritis in Canada, displaying a morphology similar to *Campylobacter* [62]. Also, it was recovered from wild rodents in China [63] and dogs in Taiwan [28]. Concerning *Helicobacter* sp. TUL, it is closely related to *Helicobacter equorum* and classified as an enterohepatic *Helicobacter* species. This novel species was named after its discovery in a febrile patient with a bloodstream infection in Caesarodunum (Tours, France) [64]. Accordingly, chicken feces may constitute an essential medium for transmitting emerging *Helicobacter* spp. where fecal droppings can directly or indirectly infect humans through water contamination. Water is a significant vehicle for the dissemination of *Helicobacter* species [2, 65], and this pathogen can persist in various environments, including soil [66, 67], raising concerns about cross-contamination between birds and the environment. Moreover, wild birds exposed to poultry excreta may transmit *Helicobacter* spp. [15, 41, 61, 68] to other birds, water sources, and new environments. In the meantime, phylogenetic analysis of the obtained *Helicobacter* sequences from layer hens in this study showed two distinct clusters. The first cluster demonstrated that *H. brantae* (PP390176), *H. kayseriensis* (PP397169), and *Helicobacter* sp. TUL (PP401975) sequences retrieved in this study were closely related to each other, implying that these *Helicobacter* spp. share a similar relationship. Furthermore, these sequences were grouped with those isolated from human cases (gastroenteritis and bloodstream infection) and environmental samples (wastewater and drinking water). In the second cluster, *H. brantae* (PP391550) was similar to *H. brantae* obtained from the feces of a resident Canada goose in the United States. *H. kayseriensis* (PP392689) exhibited a genetic relatedness to *H. kayseriensis* isolated from the feces of wild birds in Turkey. Additionally, *H. winghamensis* and *H. brantae* (PP814592 and PP814629, respectively) were grouped in the same clade and showed close relationship with *Helicobacter* sp. recovered from wild birds in Venezuela. These findings suggest that these *Helicobacter* spp. may spread from chickens to wild birds, humans, and the environment. Consequently, a comprehensive understanding of the transmission routes of *Helicobacter* infection can promote One Health approaches and facilitate the development of effective preventive strategies. The prevention and control strategies for *Helicobacter* spp., particularly *H. pullorum*, were based on the implementation of biosecurity measures in poultry farms and increasing the resistance of chickens to colonization by introducing organic acid additives to drinking water and/or feed. In addition, improved hygienic measures are required during the transport of live birds, slaughtering, and dressing of carcasses, as carcass contamination may occur through fecal matter spillage or cross-contamination [69]. Control measures should be established to reduce human exposure by minimizing the contamination of chicken meat along the food chain. Furthermore, monitoring and surveillance data would be highly crucial to mitigate the risk of *Helicobacter* infection through the implementation of One Health policies, especially in developing countries [7].

Investigation of the *cdtB* virulence gene among the *Helicobacter* species retrieved from broilers and layers in this work showed that it was present in 10 *H. pullorum*, 5 *H. brantae*, 1 *H. winghamensis*, and 1 *Helicobacter* sp. TUL; however, none of *H. kayseriensis* had *cdtB*. The *cdtB* gene appears to be the most conserved gene amongst all *cdt* genes in terms of differences between bacterial species [70]. For instance, Ceelen et al. [71] and Qumar et al. [33] detected *cdtB* in all *H. pullorum* strains obtained from poultry, while Mohamed et al. [72] observed *cdtB* in *H. pullorum* isolates from clinically healthy and diseased chickens at a prevalence rate of 32.9% and 67%, respectively. Yet, there is limited data regarding the occurrence of this virulence gene in *H. brantae*, *H. winghamensis*, *H. kayseriensis*, and *Helicobacter* sp. TUL, which requires further study. The *cdtB* is an important virulence factor that induces edema, cytoskeletal anomalies, and G2/M cycle arrest in the host cell. It causes cellular and nuclear enlargement, accompanied by profound remodelling of the actin cytoskeleton, resulting in the formation of large actin-rich cortical lamellipodia and membrane ruffle structures. Furthermore, disturbance of focal adhesion and the microtubule network were also observed. These effects may have significant consequences on bacterial adhesion and intestinal barrier integrity [22, 25]. The presence of *cdtB* in *H. pullorum* may play a significant role in various complications associated with human infections, such as gastroenteritis [22] and Crohn's disease [37]. Moreover, previous reports have shown that chronic infection by CDT-producing *H. pullorum* might lead to malignant transformation and cancer [73]. Detailed explanations of *cdtB* pathogenesis, interaction with its natural host, and factors contributing to the expression of *Helicobacter cdtB* remain unclear [21, 22, 74]. The findings of experimental infection carried out by Pratt et al. [75] suggested that CDT expression may reflect a bacterial adaptation that influences the interaction between the pathogen and the host immune system. CDT has been shown to induce apoptosis in primary human peripheral blood mononuclear cells and cultured T-cell lines [76, 77]. In addition to its direct effect on T cells, CDT may be able to interfere with immune responses via interfering with antigen-presenting cells [75]. Moreover, the bacterial adaptation of CDT production allows long-term persistence within the mammalian host and modifies the development of host immunity, resulting in specific immune responses which fail to clear the organism. In a host with an altered immune system, this modification of the specific immune response leads to the development of dysregulated immunity and colitis [75]. In this study, we provided partial sequences of *H. brantae* and *H. pullorum cdtB* from layer and broiler chickens, respectively, where these sequences exhibited a high identity percentage (98.68%-99.07%) to *H. pullorum cdtB* strains isolated from patients suffering from gastroenteritis, diarrhea, and liver cirrhosis, highlighting the public health significance of such sequences.

## Conclusion

The occurrence of emerging virulent *Helicobacter* species in broiler and layer chickens highlights the potential zoonotic role of chickens as a reservoir of *Helicobacter* infection, which raises a public health concern. Establishing zoonotic links of *Helicobacter* spp. requires a variety of ways to determine how this pathogen can be transmitted between animals and humans. There are several important methods to identify zoonotic links, including molecular and genetic analysis such as multi-locus sequence typing and whole genome sequencing; epidemiological studies like cross-sectional studies, case–control studies, surveys of animal populations, and risk factor analysis. In addition, experimental animal models should not be ruled out. When these methods are combined, they can provide compelling evidence of zoonotic linkages of *Helicobacter* species since the findings of these studies help to improve our understanding of the transmission dynamics and potential public health risks posed by these bacteria. From a One Health perspective, the interconnection between human, animal, and environmental health sectors is crucial, necessitating continuous monitoring and surveillance of *Helicobacter* infections to mitigate their public health threat.

## Data Availability

All data generated or analyzed during this study are included in this published article. The partial Helicobacter 16S rRNA gene sequences generated in this study from layer chickens were deposited in the GenBank with the following accession numbers: *Helicobacter brantae* (PP390176, PP391029, PP391658, PP391550, PP814627, and PP814629), *Helicobacter kayseriensis* (PP392689, PP397169), *Helicobacter winghamensis* (PP814592), and *Helicobacter* sp. TUL (PP401975). The accession numbers of *H. pullorum* and *H. brantae* *cdtB *gene were PP486371, and PP486372, respectively.
